# The Efficacy of Injections for Partial Rotator Cuff Tears: A Systematic Review

**DOI:** 10.3390/jcm10010051

**Published:** 2020-12-25

**Authors:** Edoardo Giovannetti de Sanctis, Edoardo Franceschetti, Ferdinando De Dona, Alessio Palumbo, Michele Paciotti, Francesco Franceschi

**Affiliations:** 1 Department of Orthopaedics and Traumatology, Catholic University, Agostino Gemelli Hospital, 00164 Rome, Italy; 2 Department of Orthopaedic and Trauma Surgery, Campus Bio-Medico University, Via Alvaro del Portillo 200, 00128 Rome, Italy; franceschetti.edo@gmail.com (E.F.); nandodedona@gmail.com (F.D.D.); alessio.palumbo@hotmail.it (A.P.); m.paciotti@unicampus.it (M.P.); 3 Department of Orthopaedic and Trauma Surgery, San Pietro Fatebenefratelli Hospital, 00189 Rome, Italy; f.franceschi@unicampus.it

**Keywords:** rotator cuff tear, conservative treatment, injections, corticosteroids

## Abstract

(1) Background: Even though rotator cuff tears are the most frequent tendon injuries in adults, the effectiveness of conservatively treated partial-thickness tears still remains a matter of debate. The purpose of this review is to compare corticosteroid injections to other drugs in the treatment of partial rotator cuff tears, focusing on the effectiveness of this therapeutic modality in terms of pain and shoulder functionality. (2) Methods: A systematic electronic search was performed using the PubMed (MEDLINE), EMBASE and Cochrane Library databases. All studies comparing the use of corticosteroids and other infiltrative techniques in the treatment of partial lesions (excluding studies that considered subacromial impingement as inclusion criteria) were pooled, data were extracted and statistically analyzed. (3) Results: Nine studies were included in this systematic review. Those studies, composed by a total of 494 shoulders treated, have been published from 1985 to 2019. All compared techniques have shown a significant improvement over baseline condition. PRP (Platelet Rich Plasma) injections have been shown to be significantly more effective in both functional and pain control only in the long term. (4) Conclusions: None of the techniques prevail indisputably on the others. Anyway, the treatment of partial rotator cuff tears with PRP injections seems to lead to significantly better outcomes in terms of pain and shoulder function in long term follow up. Whereas in short and medium term follow up, PRP injections was superior only in terms of shoulder function. The small number of studies on prolotherapy did not enable us to provide an opinion on the outcomes of this technique.

## 1. Introduction

Partial-thickness rotator cuff tears (PTRCT) are one of the most common shoulder injuries, with a prevalence of approximately 4% at age <40 years, 26% at age >60 years, and 20% in overall asymptomatic individuals [1,2,3].

A wide variety of treatment modalities have been described for those type of lesions, depending on patient’s age, activity level, symptoms, level of dysfunction, findings on physical examination and imaging [1].

Nonoperative management, including oral nonsteroidal anti-inflammatory drugs (NSAIDs), physical therapy and different types of injections [1], is considered as the first-line treatment for PTRCT, whereas surgical treatment is generally indicated in patients with conservative treatment failure after three to six months and in younger patients with traumatic tears [4].

The effectiveness of physiotherapy and strengthening muscle exercises in treating rotator cuff pathologies has been demonstrated in several systematic reviews in recent years [5,6,7,8]. In addition to physical therapies, with the aim of reducing symptoms, various infiltrative options have been developed so far [9]. Commonly, patients with an MRI showing rotator cuff disease are able to perform daily activities with no pain and disability despite the injury [10,11,12,13]. Physicians have been trying to evaluate and develop new options to better treat this shoulder disease [14]. Among those, the corticosteroids injections are the most widely used, but their use is debated due to lack of inflammation [8] in partial tendon tears and potential deleterious effects, such as tendon atrophy and decreased quality of tissue available for further repair, as demonstrated by Tillander et al [15] in animal studies. Most of the papers concerning the use of injections in rotator cuff tears focuses on corticosteroids reporting efficacy in reducing pain and improving function but with little reproducible evidence [1]. This systematic review evaluates shoulder injections in the treatment of partial rotator cuff tears, comparing corticosteroids with other drugs, focusing on the effectiveness of those treatments in terms of pain and shoulder functionality.

## 2. Experimental Section

**Figure 1:**
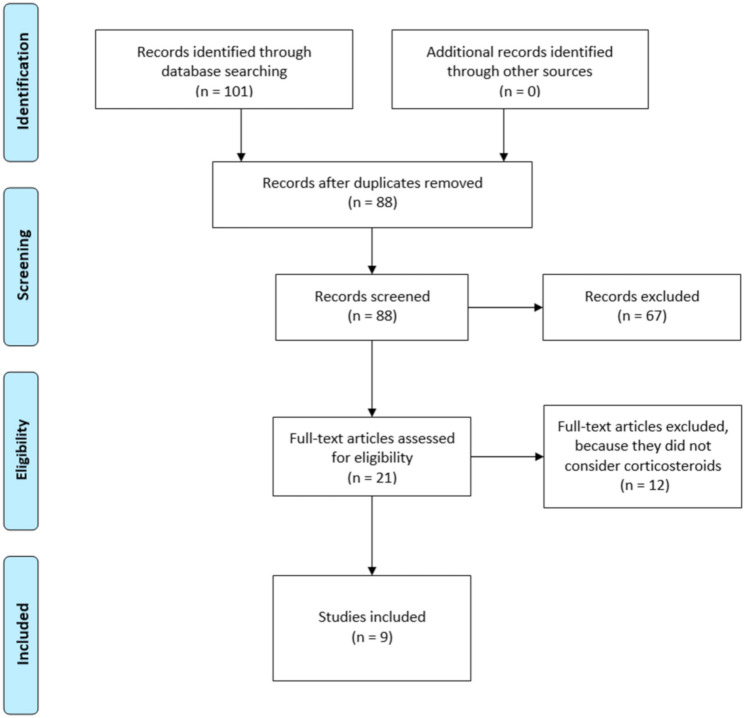
PRISMA (preferred reporting items of systematic reviews) flow diagram of study inclusion process.

This systematic review was conducted in accordance with the PRISMA guidelines (preferred reporting items of systematic reviews) [16]. The PRISMA guidelines are made up of a 27-item checklist regarding review contents and a four-phase flow diagram reporting the study selection process (Figure 1).

### 2.1. Literature Search

A comprehensive, systematic literature search was performed in July 2020. The databases of MEDLINE (PubMed), EMBASE, CINAHL (Cumulative Index to Nursing and Allied Health Literature) and the Central Registry of Controlled Trials were searched without time limits. The utilized search strings were: ((Rotator Cuff [MeSH Terms]) AND Tear [MeSH Terms]) AND conservative treatment; ((injection therapy [MeSH Terms]) AND corticosteroids [MeSH Terms]) AND partial tear. We limited the search to articles in English, and only human studies were included. All titles and abstracts were assessed by two researchers (E.F. and F.D.), and all relevant articles were obtained. All bibliographies were also hand searched to identify further relevant literature. All relevant articles were read independently in full text by two researchers to assess whether they met the inclusion criteria. If there was a difference in opinion on their suitability, a consensus was reached by consulting a third senior reviewer. Reviews and meta-analysis were also analyzed, in order to broaden the search for studies that might have been missed through the electronic search.

### 2.2. Eligibility Criteria

Only randomized or not controlled clinical trials published on peer-reviewed journals were included. Full text articles with no restriction for language were considered. Studies concerning adult patients diagnosed with rotator cuff tendinopathy/partial tear by either clinical or image evaluation were included. Whereas papers with patients treated for subacromial impingement, adhesive capsulitis, trauma, full-thickness tears, calcific rotator cuff disease, or rheumatological disease were excluded. Only studies comparing at least two injection therapy options were considered (including corticosteroid, Platelet rich Plasma, prolotherapy, placebo). The number or guidance method of injection had no restriction. Studies comparing an injection technique with physiotherapy or other noninvasive methods were excluded (Table 1).

### 2.3. Data Extraction

All the included studies were analyzed and following data were extracted and summarized in tables using Microsoft Excel (version 2013, Microsoft Corporation, Redmond, WA, USA): study type and year of publication, type of infiltration, number of patients, complications occurred, clinical scores reported. Data were extracted independently by two authors subsequently after all the eligible studies were recruited. The principal and secondary outcome of interest included respectively pain reduction and functional improvement of the shoulder (Table 2, Table 3, Table 4 and Table 5).

### 2.4. Study Quality

To evaluate the methodological quality of the included studies, MINORS (Methodological index for non-randomized studies) tool for methodological quality assessment of nonrandomized studies score [17] was assessed for each of the studies. The score contains a nine-item checklist and 12 items for comparative studies, where a score of 0–2 points is given for any single item. The score depends on the adequacy of reporting certain information, where 0 corresponded to “not reported”, 1 to “reported but inadequate” and 2 to “reported and adequate”. A total of 16 points can be achieved by a single nonrandomized or noncomparative study, while comparative studies can achieve a total of 24, since four more items are considered. Reliability was established on the basis of good inter-reviewer agreement, high test-retest reliability by the κ-coefficient and good internal consistency by a high Cronbach’s α-coefficient.

### 2.5. Statistical Analysis

Statistical analysis of the data, for the purpose of a metanalysis, was not possible due to substantial heterogeneity in study design and populations. One-way ANOVA and unpaired t-tests were applied to compare means and standard deviations of the studies analyzed.

## 3. Results

A total of 101 studies were found through the electronic searching engines. Nine studies [18,19,20,21,22,23,24,25,26] were included in this systematic review. All studies included were prospective [18,19,20,21,22,23,24,25,26,27], all of these were randomized clinical trial except one. A total of 494 shoulders were analyzed; of these 232 underwent infiltration with corticosteroids, 90 with PRP and 47 with glucose prolotherapy. The remaining patients underwent an infiltrative cycle with lidocaine or others local anesthetic as placebo.

### 3.1. Interventions

Four studies compared the usage of corticosteroids and placebo [18,19,20,21], three studies compared corticosteroids and PRP [22,23,26], one study compared corticosteroids with prolotherapy [24] and one study compared corticosteroids with all the other techniques mentioned above [26]. Each study considered different dosages for each drug. Wirington et al. compared the use of 80 mg methylprednisolone plus 2 mL of 2% lignocaine with 4 mL of 0.9% saline solution as placebo. Vecchio et al. compared the use of 40 mg methylprednisolone plus 1 mL of 1% lignocaine with 1 mL of 1% lignocaine. Alvarez et al. instead, compared 4 mL of 2% xylocaine plus 6 mg of betamethasone with 5 mL of 2% xylocaine alone. Hong et al. compared two different doses of corticosteroids with placebo: 4 mL of triamcinolone acetonide (40 mg) and 2 mL of triamcinolone acetonide (20 mg) plus 2 mL of 1% lidocaine versus 4 mL of 1% lidocaine. Von Wehren et al. and Shams et al. compared 40 mg of triamcinolone acetonide with 5 mL and 2.5 mL of autologous conditioned blood respectively. Cole et al. compared the use of 1 mL of 40 mg methylprednisolone acetate plus 1 mL of 1% lignocaine hydrochloride with 1 mL of 50% glucose (25 g/50 mL) plus 1 mL of 1% lignocaine hydrochloride giving a 25% glucose prolotherapy solution. Damjanov et al. compared 2 mL of betamethasone injection with 2 mL of autologous conditioned serum. Sari et al. were the only ones to compare all the techniques mentioned above; they compared 2 mL of 40 mg triamcinolone acetonide plus 2 mL of 1% lidocaine and 1 mL of saline, 5 mL of autologous conditioned blood plus 1 mL of 10% calcium chloride, 5 mL of a mixture of 4 mL 20% dextrose plus 1 mL of lidocaine and 5 mL of solution containing 3 mL of 1% lidocaine plus 2 mL of saline solution.

### 3.2. Outcome Measures

The principal outcomes of interest included pain reduction, which was evaluated comparing the scales used in the studies (VAS—Visual Analog Scale—scale [18,19,20,21,22,23,25,26,27] and Likert scale [24]) and functional improvement of the shoulder. Outcomes considered to assess shoulder function were: Constant Score, ASES (American Shoulder and Elbow Score), WORC (Western Ontario Rotator Cuff index), DASH (The Disability of the Arm, Shoulder and Hand), SST (Simple Shoulder Test), SDQ (Strenghts and Difficulties Questionnaire) and ROM (Range Of Motion).

### 3.3. Outcomes Data

The results have been analyzed at the various stages of follow-up: short (two to six weeks), medium (12 weeks) and long term (24 weeks and more) (Table 4).

### 3.4. Corticosteroids

Nine studies analyzed the effects of corticosteroids [18,19,20,21,22,23,24,25,26]. Five of these compared corticosteroids with placebo [18,19,20,21,26], four of these with PRP [22,23,25,26] and two with prolotherapy [24,26].

The mean values of VAS, ASES, CS, WORC and SST obtained from the analysis at the three follow up times respectively are: 2.73 ± 1.08, 2.93 ± 0.89, 4.10 ± 0.38 (VAS); 64.70 ± 6.71, 63.60 ± 4.98, 68.48 ± 11.35 (ASES); 80.70 ± 0.14, 77.50 ± 0.14, 87.40 ± 0.14 (CS); 50.52 ± 7.41, 51.22 ± 7.18, 76.45 ± 24.68 (WORC); 8.60 ± 0.14, 8.25 ± 0.07, 9.25 ± 0.07 (SST) (Table 4).

### 3.5. PRP

Four studies analyzed the effects of PRP [22,23,25,26] comparing it with corticosteroids. The mean values of VAS, ASES, CS, WORC and SST obtained from the analysis at the three follow up times respectively are: 3.51 ± 1.86, 3.90, 2.04 ± 0.76 (VAS); 63.89 ± 15.38, 76.59 ± 18.03, 76.59 ± 11.03 (ASES); 81.50 ± 0.14, 91 ± 0.14, 90.60 ± 0.14 (CS); 51.65 ± 5.79, 42.83 ± 9.63, 79.46 ± 24.09 (WORC); 8.30 ± 0.14, 10.25 ± 0.07, 10.25 ± 0.07 (SST) (Table 4).

### 3.6. Prolotherapy

Two studies analyzed the effects of prolotherapy [24,26]. The mean values of VAS, ASES and WORC obtained from the analysis are at the three follow up times respectively: 4.37 ± 1.16, 4.27 ± 1.36, 3.1 ± 1.52 (VAS); 52.6 ± 11.25, 56.1 ± 9.62, 60.37 ± 11.40 (ASES); 52.03 ± 7.79, 46.38 ± 9.01, 91.27 ± 21.79 (WORC) (Table 4).

### 3.7. Short-Term Follow-Up

The comparison of the data extrapolated from the groups showed the absence of significant differences for the VAS, ASES, WORC and SST between different types of infiltration. Nevertheless, the constant score proved to be higher for short term follow up for the PRP-treated group compared to the corticosteroid one (*p* = 0.03) (Table 4).

### 3.8. Medium-Term Follow-Up

Even in the medium-term comparison, no significant differences were found except for CS and SST. In both cases, there was a significant difference in favor of the PRP-treated group compared to the corticosteroid-treated group (*p* < 0.01; *p* < 0.01) (Table 4).

### 3.9. Long-Term Follow-Up

In the long-term comparison, the results obtained show a significant difference in favor of the PRP group compared to the other groups in terms of VAS (*p* = 0.02). Furthermore the PRP treated group had significant higher CS and SST scores than the corticosteroids treated group *(p* < 0.01; *p* < 0.01). No significant differences were found between the three groups in terms of ASES and WORC scores. (Table 4).

### 3.10. Complications

Hong et al. found the presence of transient diarrhea in one patient treated with 4 mL of triamcinolone acetonide (40 mg), facial flushing in one patient treated with 2 mL of triamcinolone acetonide (20 mg) plus 2 mL of 1% lidocaine and dizziness in one patient treated with 4 mL of 1% lidocaine. Damjanov et al. found as complications: high blood pressure (two patients), facial erythema (two patients), moon facies (one patient) and headache (three patients). All the complications highlighted by Damjanov et al. referred to the corticosteroid group with absence of complications related to the PRP group. The other studies did not report any other complications (Table 6).

### 3.11. Methodological Quality

**Figure 2:**
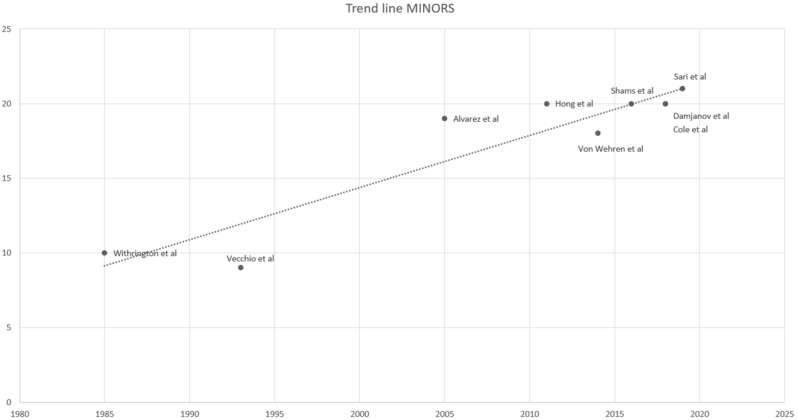
Trend line MINORS score.

The outcomes of the methodological quality assessment are shown in Table 1. An analysis of the literature has shown a progressive improvement in the quality of studies, evaluated through the use of the MINORS score. The Spearman correlation coefficient has indeed shown a significant correlation between MINORS score and the year of publication (*p* < 0.01; r = 0.87). From a graphic point of view, it is possible to notice a progressive increase in the trend line from 1985 to 2019. (Figure 2).

## 4. Discussion

The most important finding of this study was that the treatment of partial rotator cuff tears with PRP injections seems to lead to significantly better outcomes in terms of pain and shoulder function in long-term follow up, whereas, in short- and medium-term follow up, PRP injections seem to be superior only in terms of shoulder function.

Rotator cuff disorders are the most common cause of shoulder disability. However, management of rotator cuff problems remains controversial, mainly because of the remarkable variability of the clinical manifestations and insufficiency of information regarding the natural history of these disorders [28,29].

Resting, NSAIDs, physical therapies (therapeutic ultrasound, laser, tens, etc.) and training rotator cuff muscles with strengthening and stretching exercise programs are recommended for patients complaining of shoulder pain [30]. Recently, injection therapies (including steroids, PRP, prolotherapy and sodium hyaluronate) have been considered to treat those rotator cuff tendon problems. The indications are controversial and a complete agreement has not been reached so far by the various authors studying these methods [31,32]. Despite the advances in conservative treatment cases of tendinosis are still difficult to treat successfully in the long term [26]. Most of the studies did not include a detailed description of patient randomization methods; thus, we could not effectively evaluate for patient selection bias. Using ROM as a surrogate outcome measure for functional assessment data should be viewed with caution, since the possible differences found could be due to inter- and intraoperator variability.

The use of corticosteroids should be carefully evaluated given the high risk of muscle weakness, tendon rupture and collagen collapse [33]. PRP is a method recently developed due to the discovery of growth factors released by platelets, which have been shown to be effective in tissue repair. Prolotherapy injection is a technique that has been previously used treating other orthopedic diseases; the ease of application, the reduced cost and the reduction of the rehabilitation process make it advantageous [34].

This paper revealed that, with the aim of reducing pain, the effect of corticosteroid injection is stronger in the short and medium term compared to other injections although not statistically significant, whereas PRP provided better functional outcomes during the whole follow up period analyzed and more pain reduction in the long term (Table 4). Therefore, for patients with partial rotator cuff tear, corticosteroid plays a role in the short term but not in long-term pain reduction. By contrast, PRP may yield better outcomes according to shoulder functionality and long-term pain reduction. Only one study compared PRP and prolotherapy over the long term as regards pain control, highlighting the absence of significant differences [26].

The short-term “efficacy” of corticosteroids as found in this review is in agreement with previous evidence [8,9,35]. Several systematic reviews have found the effectiveness of corticosteroids in the treatment of shoulder disorders [36,37,38,39,40,41].

Several authors agree with the concept that repeated corticosteroids injections at short intervals are dangerous with regard to tendon atrophy and reduction of connective tissue quality [42,43]. Despite the efficacy of prolotherapy on rotator cuff lesions [8], tendinopathies and fasciopathies of the lower limbs [44] reported in different papers, only two studies included in this review analyzed this technique; therefore more comparative trials need to be carried out to better evaluate this treatment.

The complications reported were described in only two of the studies analyzed [21,25]. Hong et Al. reported no serious complications other than transient diarrhea on day 3 after injection, facial flushing on days and dizziness due to vasovagal reaction during injection in the [21] Damjanov et al. noticed eight transient adverse events (AEs) in three patients within the corticosteroids treated group, listed as headache, arterial hypertension, facial erythema and facies lunata. No AEs were reported in the PRP group during the 24-week follow up period [25].

The remaining studies did not find or did not report any complications, although several studies in the literature have described tendon rupture events associated with the use of corticosteroids [45]. Because of the heterogeneity of the studies, a meta-analysis was not conducted. The available data does not allow to calculate the frequency and the optimal number of infiltrations to be carried out.

### Strengths and Limitations

Despite the PRP group constant score being significantly higher in short and long term follow up, the difference with the corticosteroid group was below the MCID (minimal clinical importance difference). Therefore, our results should be taken with caution, as the PRP may not be the appropriate treatment for every partial tear. It may be possible that a clinical difference would present with a longer follow-up period.

An inter-reviewer agreement in assessing MINORS score of the studies included was not calculated, which is a limitation of the review process. The most relevant limitation of the present investigation is the low number of studies available on this topic. A further limitation is the difficulty to compare different outcomes, which was related to the differences in study design and in dose and medication used for the treatment. Moreover, there is heterogeneity in diagnosis criteria among different trials. Many of the trials used clinical diagnosis for rotator cuff tendinopathy without image confirmations and it may be very difficult to differentiate a partial rupture from a total one. However, the present systematic review is the first piece of work carried out with the aim to evaluate the role of conservative treatment for partial thickness rotator cuff tears.

## 5. Conclusions

None of the techniques completely outperforms the others; a statistically significant improvement compared to baseline was found in all the surveys carried out for all the procedures. There are no statistically significant differences in terms of pain control between the treatment analyzed in short- and medium-term follow up. PRP injections seems to show significant long-term superiority (even if with a CS difference below the MCID) over other methods investigated. In terms of shoulder function, the PRP was better in all follow-ups considered. No clear consensus can be found on the frequency of injections.

The small number of studies analyzed regarding prolotherapy prevented our evaluating this treatment in depth. Future RCTs to better delineating the role of subacromial injection using different types of drugs in the management of partial rotator cuff tears are needed.

## Figures and Tables

**Table 1:** Study details of the included articles. LOE, level of evidence; RCT, randomized clinical trial; CT, clinical trial; RC, rotator cuff; MRI, magnetic resonance imaging.

Study	LOE	Study	Year	Procedures	Inclusion Criteria	No. of Patients (Shoulders)	Disease Stage	MINORS Score
Withrington et al. [18]	I	RCT	1985	Corticosteroid vs. placebo	Clinical supraspinatus tendonitis	12(12)/13(13)	-	10
Vecchio et al. [19]	I	RCT	1993	Corticosteroid vs. placebo	Clinical diagnosed acute RC tendinitis	28(28)/27(27)	-	9
Alvarez et al. [20]	I	RCT	2005	Corticosteroid vs. placebo	Chronic tendinosis or partial cuff tear	30(30)/28(28)	-	19
Hong et al. [21]	I	RCT	2011	Corticosteroid (double dose) vs. corticosteroid vs. placebo	Clinically or ultrasound diagnosed RC lesion	27(27)/25(25)/27(27)	-	20
Von Wehren et al. [22]	III	CT	2014	Corticosteroid vs. PRP	MRI evidence of partial supraspinatus tear	25(25)/25(25)	II-III-IV	18
Shams et al. [23]	I	RCT	2016	Corticosteroid vs. PRP	Painful partial RC tears diagnosed by MRI	20(20)/20(20)	II-III-IV	20
Cole et al. [24]	I	RCT	2018	Corticosteroid vs. Prolotherapy	Ultrasound evidence of supraspinatus tendinopathy	19(19)/17(17)	-	20
Damjanov et al. [25]	I	RCT	2018	Corticosteroid vs. ACS	Ultrasonography evidence of supraspinatus tendinopathy	16(16)/15(15)	-	20
Sari et al. [26]	I	RCT	2019	Corticosteroid vs. PRP vs. Prolotherapy vs. placebo	RC pathology (bursitis, RC tendinosis, or partial tears)	30(30)/30(30)/ 30(30)/30(30)	-	21

**Table 2:** Main outcomes. Abbreviations: ASES, American Shoulder and Elbow Surgeons Shoulder Score; DASH, Disabilities of the Arm, Shoulder and Hand Score; ROM, range of motion; Rx, treatment; SDQ, Shoulder Disability Questionnaire; SST, Simple Shoulder Test; VAS, visual analog scale; WORC, Western Ontario Rotator Cuff Index; CS, Constant Shoulder Score; wk, weeks.

Study	Follow-Up (wk)	Outcome Measure	Procedures/Rx Dose
Withrington et al. [18]	2, 8	VAS, paracetamol count	Steroid vs. Placebo
Vecchio et al. [19]	2, 4, 12	VAS, ROM	Steroid vs. Placebo
Alvarez et al. [20]	2, 6, 12, 24	VAS, DASH, ASES, WORC, ROM	Steroid vs. Placebo
Hong et al. [21]	2, 4, 8	VAS, SDQ, ROM	Steroid (double dose) vs. Steroid vs. Placebo
Von Wehren et al. [22]	6, 12, 24	VAS, CSS, ASES, SST	Steroid vs. PRP
Shams et al. [23]	6, 12, 24	VAS, CSS, ASES, SST	Steroid vs. PRP
Cole et al. [24]	6, 12, 24	Pain calculated with a 5 pt Likert scale, ROM	Steroid vs. Prolotherapy
Damjanov et al. [25]	0, 4, 24	VAS, CSS	Steroid vs. PRP
Sari et al. [26]	3, 12, 24	VAS, ASES, WORC	Steroid vs. PRP vs. Prolotherapy vs. Placebo

**Table 3:** Summary: pain-function scores. N.a., not available, data reported by the article do not permit the evaluation of the parameter; ^a^ sum of visual analogue scores for pain at rest, movement and night; † scored on a five-point Likert scale (very bad, bad, poor, fair, good) and converted to a numerical score from 0 to 4 (not VAS); †† CSS ( Constant Shoulder Score) scores: <11 is excellent, 11–20 is good, 21–30 is fair and >30 is poor; * SST; ** WORC. Abbreviations: ASES, American Shoulder and Elbow Surgeons Shoulder Score; CS, Constant Shoulder Score; WORC, Western Ontario Rotator Cuff Index; SST, Simple Shoulder Test; Pre: preoperative; Post: postoperative at last follow-up.

Study	Follow-up (wk)	Procedures/Rx Dose	VAS for Pain		ASES		CS		WORC/SST	
			Pre	Post	Pre	Post	Pre	Post	Pre	Post
**Withrington et al.** [18]	2, 8	Steroid: 80 mg methylprednisolone plus 2 mL of 2% lignocaine	2.72 variation							
		Placebo: 4 mL 0.9% saline	1.16 variation							
**Vecchio et al.** [19]	2, 4, 12	Steroid: 40 mg methylprednisolone plus 1 mL of 1% lignocaine	14 (12–19) ^a^	8 decrease						
		Placebo: 1% lignocaine 1 mL	15 (12–18) ^a^	8 decrease						
**Alvarez et al.** [20]	2, 6, 12, 24	Steroid: 4 mL of 2% xylocaine plus 6 mg of betamethasone	54.8 (0–100)	45.2 (0–100)	46.9 ± 18.3	62.3 ± 22.9			38.1 ± 17 **	59 ± 26 **
		Placebo: 5 mL of 2% xylocaine alone	61.9 (0–100)	42.6 (0–100)	41.7 ± 16	60.4 ± 24.2			35.4 ± 19 **	51 ± 32 **
**Hong et al.** [21]	2, 4, 8	Steroid: 4 mL of triamcinolone acetonide (40 mg)	5.5 ± 1.8	2.0 ± 2.3						
		Steroid: 2 mL of 20 mg of triamcinolone acetonide + 2 mL of 1% lidocaine	6.0 ± 1.4	3.2 ± 1.9						
		Placebo: 4 mL of 1% lidocaine	5.3 ± 1.6	4.7 ± 2.2						
**Von Wehren et al.** [22]	6, 12, 24	Steroid: 40 mg triamcinolone acetonide	N.a.	N.a.	50.6 ± 14	82.5 ± 25.4	69.9 ± 19.5	87.5 ± 12.3	5.8 ± 3.2 *	9.3 ± 2.6 *
		PRP: 5 mL of autologous conditioned blood	N.a.	N.a.	50.7 ± 15	77.1 ± 19.3	66.2 ± 21.1	90.7 ± 9.4	6.5 ± 3.1 *	10.3 ± 2.1 *
**Shams et al.** [23]	6, 12, 24	Steroid: 40 mg triamcinolone acetonide	N.a.	N.a.	52.5 ± 15	78.9 ± 13.2	69.7 ± 19.4	87.3 ± 12.2	5.6 ± 3.1 *	9.2 ± 2.7 *
		PRP: 2–2.5 mL of autologous conditioned blood	N.a.	N.a.	52.6 ± 16	83.4 ± 16.1	66 ± 21	90.5 ± 8.3	6.3 ± 3 *	10.2 ± 1.8 *
**Cole et al.** [24]	6, 12, 24	Steroid: 1 mL of 40 mg/mL methylprednisolone acetate plus 1 mL of 1% lignocaine hydrochloride	1.8 †	2.4†						
		Prolo: 1 mL of 50% glucose (25 g/50 mL) + 1 mL of 1% lignocaine hydrochloride giving a 25% glucose prolotherapy solution.	1.9 †	2.8 †						
**Damjanov et al.** [25]	0, 4, 24	Steroid: 2 mL of bethametasone injection	65	40			87.5% poor; 12.5% fair; ††	53.3% poor; 13.3% fair; 20% good; 13.3% excellent; ††		
		PRP: 2 mL of autologous conditioned serum	70	15			86.7% poor; 13.3% excellent; ††	6.7% poor-fair-good; 80% excellent; ††		
**Sari et al.** [26]	3, 12, 24	Steroid: 2 mL of 40 mg triamcinolone acetonide plus 2 mL 1% lidocaine and 1 mL saline.	5.63 ± 0.93	3.77 ± 1.41	40.13 ± 8.18	55.63 ± 11			51.4 ± 7.73 **	93.90 ± 17.94 **
		PRP: 5 mL of autologous conditioned blood plus 1 mL 10% calcium chloride	5.63 ± 1.00	2.57 ± 1.19	46.28 ± 8.61	63.87 ± 11.96			50.79 ± 6.48 **	79.46 ± 24.09 **
		Prolotherapy: 5 mL of a mixture of 4 mL 20%dextrose and 1 mL lidocaine	5.9 ± 0.88	3.1 ± 1.52	45 ± 9.42	60.37 ± 11.4			53.67 ± 8.43 **	91.27 ± 21.79 **
		Placebo: 5 mL solution containing 3 mL 1% lidocaine plus 2 mL saline solution	5.47 ± 0.86	3.2 ± 1.19	47.27 ± 7.44	47.27 ± 7.44			52.13 ± 7.92 **	96.55 ± 20.43 **

**Table 4:** Clinical Outcomes. Bold characters denote significant values. * *p* < 0.05.

Score		Corticosteroids	PRP	Prolotherapy	*p* Value (ANOVA)
**VAS**	Pre-op.	5.6 ± 0.66	6.2 ± 1.2	5.3 ± 0.81	0.56
	Short term	2.73 ± 1.08	3.51 ± 1.86	4.37 ± 1.16	0.19
	Mid term	2.93 ± 0.89	3.9	4.27 ± 1.36	0.13
	Long term	4.09 ± 0.38	2.04 ± 0.76	3.1 ± 1.52	0.02 *
**ASES**	Pre-op.	48 ± 5.5	50 ± 3.2	45 ± 9.42	0.65
	Short term	64.70 ± 6.71	63.89 ± 15.38	52.60 ± 11.25	0.36
	Mid term	63.60 ± 4.98	76.59 ± 18.03	56.10 ± 9.62	0.18
	Long term	68.48 ± 11.35	76.59 ± 11.03	60.37 ± 11.40	0.28
**COSTANT**	Pre-op.	69 ± 2.8	68 ± 2.5	-	0.89
	Short term	80.70 ± 0.14	81.50 ± 0.14	-	0.03 *
	Mid term	77.50 ± 0.14	91 ± 0.14	-	<0.01 *
	Long term	87.40 ± 0.14	90.60 ± 0.14	-	<0.01 *
**WORC**	Pre-op.	45 ± 9.4	51 ± 6.48	54 ± 8.43	0.77
	Short term	50.52 ± 7.41	51.65	52.03 ± 7.79	0.06
	Mid term	51.22 ± 7.18	42.83	46.38 ± 9.01	0.27
	Long term	76.45 ± 24.68	79.46	91.27 ± 21.79	0.60
**SST**	Pre-op.	6.2 ± 0.49	6.0 ± 0.49	-	0.72
	Short term	8.6 ± 0.14	8.3 ± 0.14	-	0.16
	Mid term	8.25 ± 0.07	10.25 ± 0.07	-	<0.01 *
	Long term	9.25 ± 0.07	10.25 ± 0.07	-	<0.01 *

**Table 5:** Summary: ROM. N.a. not available, data reported by the article do not permit the evaluation of the parameter; * median/(interquartile range); ** differences between medians.

Study	Procedures	Active abduction (°)		Active Forward Flexion (°)		Active External Rotation (°)		Active Internal Rotation (°)	
		Pre	Post (Last Follow-Up)	Pre	Post (Last Follow-Up)	Pre	Post (Last Follow-Up)	Pre	Post (Last Follow-Up)
Withrington et al. [18]	Steroid	64.6°	N.a.						
	Placebo	61.9°	N.a.						
Vecchio et al. [19]	Steroid	155 (105-180) *	0 (-10-50) **			45 (15-55) *	0("5-^0) **		
	Placebo	160(130-180) *	0 (0-20) **			40 (20-60) *	20(0-40) **		
Alvarez et al. [20]	Steroid			138.9° ± 23.7°	139.0° ± 21.8°	75.3° ± 16°	75.7° ± 23.6°	45.3° ± 23°	46.4° ± 24°
	Placebo			136.3° ± 28.8°	143.7° ± 27.8°	80.4° ± 26.5°	63.7° ± 25°	40.9° ± 30°	49.2° ± 27.4°
Hong et al. [21]	Steroid (double dose)	141.7° ± 27.4°	161.1° ± 25.0°	153.0° ± 17.3°	164.9° ± 15.7°	64.3° ± 16.9°	84.6° ± 15.2°	44.0° ± 14.7°	64.7° ± 15.0°
	Steroid	137.8° ± 26.8°	162.7° ± 20.6°	151.7° ± 19.1°	163.0° ± 16.9°	59.0° ± 16.6°	84.6° ± 11.9°	42.7° ± 19.3°	59.7° ± 19.3°
	Placebo	140.7° ± 21.4°	137.6° ± 21.1°	155.4° ± 12.3°	157.9° ± 13.5°	63.7° ± 18.5°	63.9° ± 23.0°	40.7° ± 13.3°	41.9° ± 14.4°
Von Wehren et al. [22]	Steroid								
	PRP								
Shams et al. [23]	Steroid								
	PRP								
Cole et al. [24]	Steroid	153°	163°	161°	165°	60°	63°		
	Prolotherapy	166°	175°	167°	172°	67°	61°		
Damjanov et al. [25]	Steroid								
	PRP								
Sari et al. [26]	Steroid								
	PRP								
	Prolotherapy								
	Placebo								

**Table 6:** Complications.

Study	Follow-Up (wk)	No. of Patients (Shoulders)	Complications
Withrington et al. [18]	2, 8	12(12)/13(13)	No mention
Vecchio et al. [19]	2, 4, 12	28(28)/27(27)	No mention
Alvarez et al. [20]	2, 6, 12, 24	30(30)/28(28)	No mention
Hong et al. [21]	2, 4, 8	27(27)/25(25)/ 27(27)	***Steroid*** (***x2***): Transient diarrhea (1)
			***Steroid***: Facial flushing (1)
			***Placebo***: Dizziness (1)
Von Wehren et al. [22]	6, 12, 24	25(25)/25(25)	No infection reported
Shams et al. [23]	6, 12, 24	20(20)/20(20)	No infection reported
Cole et al. [24]	6, 12, 24	19(19)/17(17)	No mention
Damjanov et al. [25]	0, 4, 24	16(16)/15(15)	***Steroid***: Arterial hypertension (2); Facial erythema (2); Facies lunata (1); Headache (3); Total = 8 AE;
			***PRP***: No complications reported
Sari et al. [26]	3, 12, 24	30(30)/30(30)/ 30(30)/30(30)	No mention

## Data Availability

Data derived from public domain resources.
